# The *Croatian Medical Journal* over three decades: the impact beyond the impact factor

**DOI:** 10.3325/cmj.2022.63.405

**Published:** 2022-10

**Authors:** Hrvoje Barić, Jelka Petrak, Nataša Kovačić, Svjetlana Kalanj Bognar, Croatian Medical Journal, Zagreb, Croatia, Department of Neurosurgery, University Hospital Center Zagreb, Zagreb, Croatia, University of Zagreb, School of Medicine, Zagreb, Croatia

Much has changed in the thirty years since the launching of the *Croatian Medical Journal* (CMJ); yet, some constants prevail. The Journal is a war child, born among ruined infrastructure, during air raids, amid uncertainty and chaos. It stood for something far greater than a scholarly journal. It was a materialization of a collective striving toward a better future and an act of defiance. The guiding spirit behind its coming to life was the same one that bolstered Croatian sports and cultural achievements and that ultimately catalyzed social and political changes. In other words, the Journal arose from a community wider than the medical one and it disseminated ideas that reverberated beyond the scientific community.

Since day one, the founders envisioned the Journal not only as a scientific communication platform and a vehicle of knowledge dissemination, but as an educational hub for future editors, reviewers, scientists, and competent medical professionals. This was (and is) reflected in the editorial policy aimed at educating local and regional medical professionals on the principles of scientific research and communication (1,2). Over the years, the Journal staff has educated cohorts of students and medical doctors on scientific methodology through undergraduate and postgraduate courses, seminars, and lectures (3). Pursuing this strategy has paved the way for achieving one of the main goals of the *CMJ* – to serve as a gateway between Croatian medical researchers and the global scientific community. This translated into more than 40% entries by Croatian authors out of more than 2700 articles published so far in the *CMJ* covered by Web of Science and Scopus. Notably, articles from Croatian authors and authors from neighboring and regional countries together account for more than half of the journal contributions (Croatia 1208, Slovenia 125, Bosnia and Herzegovina 88, Serbia 86, Northern Macedonia 5, Hungary 44, Italy 59). These numbers illustrate a remarkable role of the C*MJ* in medical publishing in the regional context. Likewise, the Journal has been striving to educate local and regional researchers on the principles of good peer-review by recruiting them into its pool of peer-reviewers, acknowledging their contribution, and helping them grow as reviewers (4). The fruit of these efforts is the increasing proportion of national and regional reviewers providing high-quality reviews. Undeniably, in many ways the Journal has served as a role model for newly emerging national and regional journals. It has done so by adopting and promoting international principles of good publishing practice. For example, the *CMJ* was the first scientific journal in Croatia to employ a research integrity editor and one of the rare journals in the 1990s to have a statistical editor among its ranks. In this sense, the guiding principles of the Journal have not changed much (5).

In the new millennium, the social environment profoundly changed. Those who occupied the scientific publishing landscape dominated by hard copies, enveloped submissions, and dial-up speeds (if any) could have hardly imagined the today’s almost exclusively virtual niche populated by social networks, alternative metrics, and predatory publishing. To keep pace, the Journal has adapted to the selection pressures on various levels. Trivial and technical amendments, such as the new web design and submission interface, were implemented, but also major policy directives regarding issues such as Journal ownership and autonomy, business and publishing model, and author rights (6). Again, the principles of innovativeness and responsiveness to the ever changing landscape have prevailed. The Journal has been endorsing international publishing standards and ethical principles, and its editors have been actively involved in major international associations such as International Committee of Medical Journal Editors, World Association of Medical Editors, and European Association of Science Editors.

Today, thirty years after the Journal launching, blood has again been spilled on the European soil, and we are facing a future filled with unknowns and uncertainty. As the society grows more interconnected, problems become more global, immediate, and existential – from pandemics to climate change and culture wars. We remain vigilant about these universal societal issues affecting public health and well-being (7-9). Yet among the turmoil, there still is a place for a constant embodied by the *CMJ*. This constant is communication – honest, unbiased, open-access, and widely available communication between researchers and the general public, physicians and patients, mentors and students. This communication should be about not only medical science but about broader topics as well. The field of medicine and its practitioners do not exist in a vacuum (and neither do any other professionals) but depend on the entirety of social factors and get increasingly specialized. For this reason, communication is essential for interdisciplinary cohesion and serves to bond and guide us forward. We believe that the *CMJ* has been a reliable partner and mediator in this communication, and that the products of our labor reach far beyond the journal impact factor.
